# 

**Published:** 1928

**Authors:** 


### CONTENTS.

| | Page |
|---|---|
| The Comparative Value of Radiation Therapy in Dermatology. By Ernest Dore, M.D., F.R.C.P. .. .. .. .. .. | 85 |
| The Evidences of Heart Failure, and the Signs and Symptoms of Three Cases personally examined. By P. Christine Vine | 97 |
| Ocular Manifestations following Encephalitis Lethargica. By A. Michael Critchley, M.B., Ch.B. .. .. .. .. .. | 113 |
| A Case of Mitral Stenosis with Auricular Fibrillation, treated with Digitalis by the Cary Eggleston Method. By K. H. Pridie .. .. .. .. .. .. .. .. | 125 |
| Sugar Tolerance Tests. By Hugh D. Pyke .. .. .. .. | 129 |
| A Note on the Pathology of Erythema Nodosum. By A. C. Fisher .. .. .. .. .. .. .. .. | 137 |
| Reviews of Books .. .. .. .. .. .. .. .. | 143 |
| Editorial Notes .. .. .. .. .. .. .. .. | 151 |
| Obituary: John Percy Ingham Harty, F.R.C.S. .. .. .. | 155 |
| Meetings of Societies .. .. .. .. .. .. .. | 158 |
| The Medical Library of the University of Bristol .. .. | 159 |
| Local Medical Notes .. .. .. .. .. .. .. .. | 162 |

